# Natriuretic peptides promote glucose uptake in a cGMP-dependent manner in human adipocytes

**DOI:** 10.1038/s41598-018-19619-0

**Published:** 2018-01-18

**Authors:** Marine Coué, Valentin Barquissau, Pauline Morigny, Katie Louche, Corinne Lefort, Aline Mairal, Christian Carpéné, Nathalie Viguerie, Peter Arner, Dominique Langin, Mikael Rydén, Cedric Moro

**Affiliations:** 1 INSERM, UMR1048, Institute of Metabolic and Cardiovascular Diseases, Obesity Research Laboratory, Toulouse, France; 2 University of Toulouse, UMR1048, Paul Sabatier University, Toulouse, France; 3 INSERM, UMR1048, Team 1, Institute of Metabolic and Cardiovascular Diseases, Toulouse, France; 4 Department of Medicine-H7, Karolinska Institutet, Karolinska University Hospital, Stockholm, Sweden; 5 Department of Clinical Biochemistry, Toulouse University Hospitals, Toulouse, France

## Abstract

Robust associations between low plasma level of natriuretic peptides (NP) and increased risk of type 2 diabetes (T2D) have been recently reported in humans. Adipose tissue (AT) is a known target of NP. However it is unknown whether NP signalling in human AT relates to insulin sensitivity and modulates glucose metabolism. We here show in two European cohorts that the NP receptor guanylyl cyclase-A (GC-A) expression in subcutaneous AT was down-regulated as a function of obesity grade while adipose NP clearance receptor (NPRC) was up-regulated. Adipose *GC-A* mRNA level was down-regulated in prediabetes and T2D, and negatively correlated with HOMA-IR and fasting blood glucose. We show for the first time that NP promote glucose uptake in a dose-dependent manner. This effect is reduced in adipocytes of obese individuals. NP activate mammalian target of rapamycin complex 1/2 (mTORC1/2) and Akt signalling. These effects were totally abrogated by inhibition of cGMP-dependent protein kinase and mTORC1/2 by rapamycin. We further show that NP treatment favoured glucose oxidation and *de novo* lipogenesis independently of significant gene regulation. Collectively, our data support a role for NP in blood glucose control and insulin sensitivity by increasing glucose uptake in human adipocytes. This effect is partly blunted in obesity.

## Introduction

Atrial- and B-type Natriuretic Peptides (NP), ANP and BNP respectively, are well-known cardiovascular hormones produced by the right atria of the heart in response to mechanical stretch. They signal through the guanylyl cyclase-A (GC-A), a transmembrane receptor exhibiting guanylyl cyclase activity^1–4^. ANP and BNP can also bind to a clearance receptor named NPRC that sequesters, internalizes and degrades the peptides^4^. Over the last decade, NP have emerged as potent metabolic hormones as recently discussed^5–8^. NP were first identified as potent lipolytic hormones in human adipocytes^9^. They signal through the second messenger cGMP and downstream activation of a cGMP-dependent protein kinase-I (PRKGI)^10^. NP have subsequently been shown to modulate adipokine secretion^11^, and the browning of white fat cells^12^.

Several cohort and community-based studies have reported a strong association between plasma NP levels and obesity. In 2004, Thomas Wang and coworkers showed an inverse relationship between plasma NP levels and body mass index (BMI)^13^, findings which were then confirmed in several independent studies^14,15^. Kahn *et al*. latter demonstrated an inverse relationship between plasma NP levels, insulin resistance and fasting blood glucose^16^. More recently, at least three prospective studies demonstrated a robust association between baseline plasma NP concentrations and the incidence of new onset type 2 diabetes (T2D)^17–19^. However a causal link between reduced plasma NP levels in obesity, insulin resistance and T2D has not yet been demonstrated. Thus it is so far unclear how NP may influence blood glucose control in humans.

Considering that adipose tissue is a major target organ of NP^20–22^ and that glucose metabolism in adipocytes is a determinant of whole-body insulin sensitivity^23,24^, we hypothesized that NP signalling in adipose tissue could be a determinant of insulin sensitivity and blood glucose control. The aim of the present study was: 1) to explore the relationships between adipose NP receptor expression and obesity, insulin resistance and T2D, and 2) to determine the impact of NP on glucose metabolism in human adipocytes. Our data demonstrate a robust link between adipose GC-A expression and insulin sensitivity, and highlight a novel biological pathway in human adipocytes by which NP promote glucose uptake and metabolism in a cGMP-dependent manner.

## Results

### Adipose NP receptors expression is altered in obesity and type 2 diabetes

**Figure 1:**
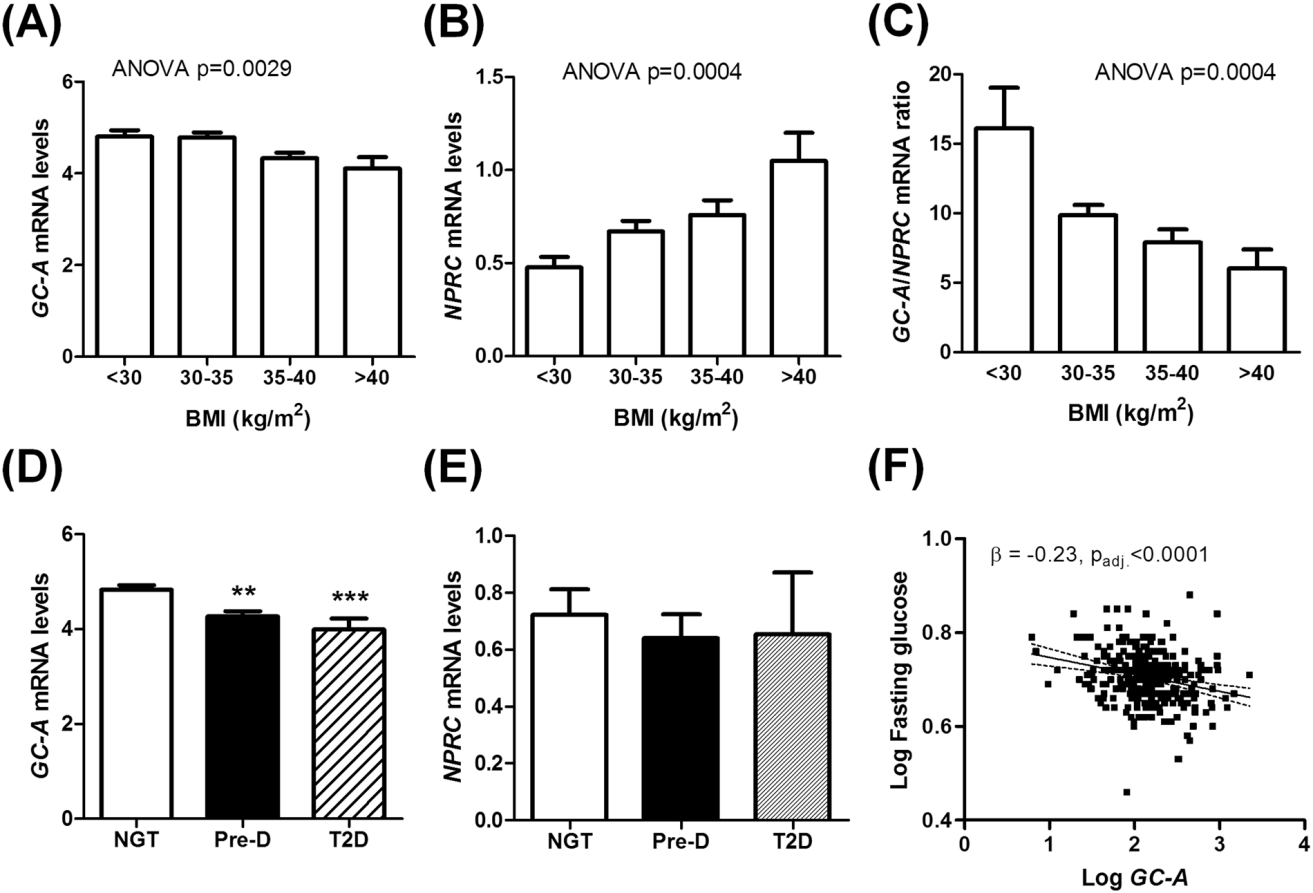
Natriuretic peptide receptor expression in human adipose tissue in obesity and type 2 diabetes. Human adipose tissue gene expression of *GC-A* (**A**), *NPRC* (**B**), and the ratio of *GC-A*-to-*NPRC* (**C**) as a function of the obesity class. Human adipose *GC-A* (**D**) and *NPRC* (**E**) mRNA levels in subjects with normal glucose tolerance (NGT), prediabetes (Pre-D) and type 2 diabetes (T2D). (**F**) Relationship between human adipose *GC-A* gene expression and fasting blood glucose (n = 311 from cohort 1). **p < 0.01, ***p < 0.0001 vs. NGT (n = 33–144 per group from cohort 1).

*GC-A* and *NPRC* gene expression was investigated in human adipose tissue biopsy samples from Cohort 1. We observed a gradual down-regulation of adipose *GC-A* mRNA levels as a function of the obesity grade, with the lowest expression levels in subjects with BMI >40 kg/m^2^ (Fig. 1A). In contrast, adipose *NPRC* mRNA levels were progressively higher as a function of BMI and were nearly doubled in subjects with BMI >40 kg.m^−2^ (Fig. 1B). Thus the ratio of *GC-A*-to-*NPRC* gene expression was significantly reduced by 39% for BMI between 30 and 35 kg.m^−2^, and by 63% for BMI >40 kg.m^−2^ (Fig. 1C). We also found a significant decrease of adipose *GC-A* expression in prediabetic (PreD, defined as impaired fasting glucose or glucose tolerance) and type 2 diabetic subjects (T2D) compared to individuals with normal glucose tolerance (NGT) (Fig. 1D), while no change was observed for *NPRC* (Fig. 1E). This association was independent of BMI as the average BMI was comparable between groups (NGT: 34.0 ± 0.3, Pre-D: 34.2 ± 0.4, T2D: 34.5 ± 1.3 kg.m^−2^). Adipose *GC-A* mRNA level was also reduced with increasing quartiles of HOMA-IR, demonstrating that the most insulin resistant individuals have the lowest adipose *GC-A* gene expression (Supplemental Fig. 1A), while no significant change was observed for *NPRC* (Supplemental Fig. 1B). In multivariate regression analyses, adipose *GC-A* levels correlated negatively with HOMA-IR (r = −0.20, p = 0.008), even after adjustment for BMI (β = −0.123, p_adj._ = 0.031). Finally, we observed a significant negative correlation (r = −0.26, p < 0.0001) between adipose *GC-A* gene expression and fasting blood glucose at baseline that was independent of BMI (β = −0.229, p_adj._ < 0.0001) (Fig. 1F). These data demonstrate a strong link between adipose NPR expression, obesity and blood glucose control.

### Adipose GC-A expression relates to insulin sensitivity

**Figure 2:**
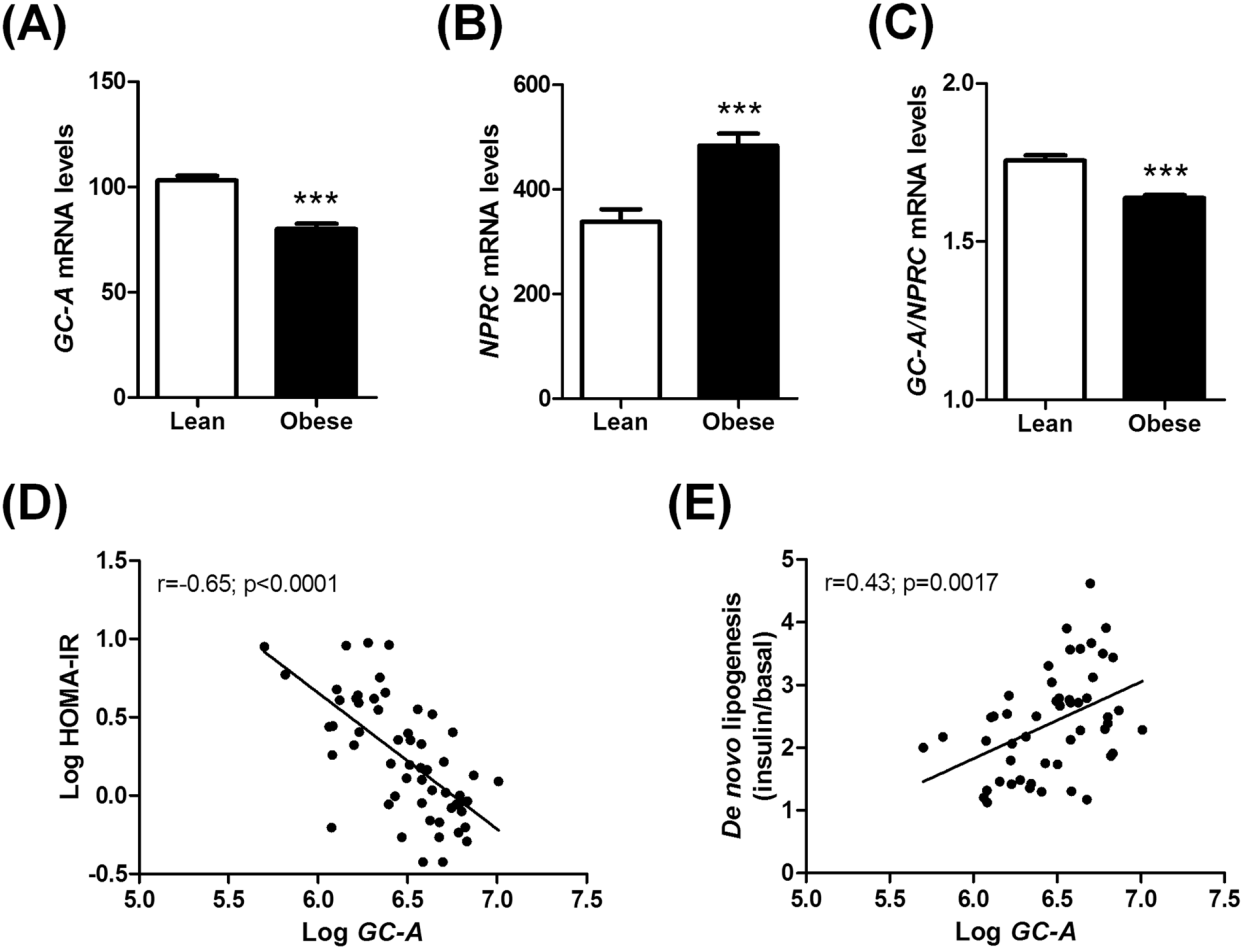
Human adipose tissue gene expression from microarrays of *GC-A* (**A**), *NPRC* (**B**), and the ratio of *GC-A*-to-*NPRC* (**C**) in lean versus obese individuals from cohort 2 (n = 56). Correlation between adipose *GC-A* expression and HOMA-IR (**D**) and *de novo* lipogenesis measured in isolated adipocytes (**E**). ***p < 0.0001 vs. lean.

The findings in cohort 1 were validated in cohort 2 showing a reduced adipose *GC-A* expression (Fig. 2A), increased *NPRC* expression (Fig. 2B) and reduced *GC-A*-to-*NPRC* ratio (Fig. 2C) in obese compared to lean individuals. Moreover, a strong inverse relationship between adipose *GC-A* mRNA levels and HOMA-IR was noted (Fig. 2D). As whole-body insulin sensitivity has been linked to adipose *GLUT4* and *MLXIPL* (encoding chREBP) expression^23,24^, we studied the relationships between these two genes and *GC-A* in cohort 2. *GC-A* was positively correlated with both *MLXIPL* (Supplemental Fig. 2A) and *GLUT4* (Supplemental Fig. 2B) mRNA levels in adipose tissue. Importantly, the correlation between *GC-A*, HOMA-IR, *GLUT4* and *MLXIPL* remained very significant even after statistical adjustment for BMI (Table 1). These robust associations were largely confirmed in cohort 1 (Supplemental Fig. 2C,D). Interestingly, we further noticed in cohort 2 a significant association between adipose *GC-A* expression and *de novo* lipogenesis, a major pathway for glucose disposal, measured in isolated adipocytes (Fig. 2E). Overall, our data indicate that adipose GC-A may be involved in the regulation of glucose metabolism and whole-body insulin sensitivity in a cell-autonomous manner.

**Table 1:** Correlations between adipose *GC-A* gene expression and HOMA-IR, and adipose *GLUT4* and *MLXIPL* (ChREBP) gene expression after adjustment for BMI.

Parameter	***GC-A***		**BMI**	
	Partial r	p value	Partial r	p value
HOMA-IR	−0.38	0.007	0.39	0.006
*GLUT4*	0.57	<0.0001	−0.18	0.19
*MLXIPL*	0.57	0.0002	−0.13	0.37

### Natriuretic peptides promote glucose uptake in human adipocytes

**Figure 3:**
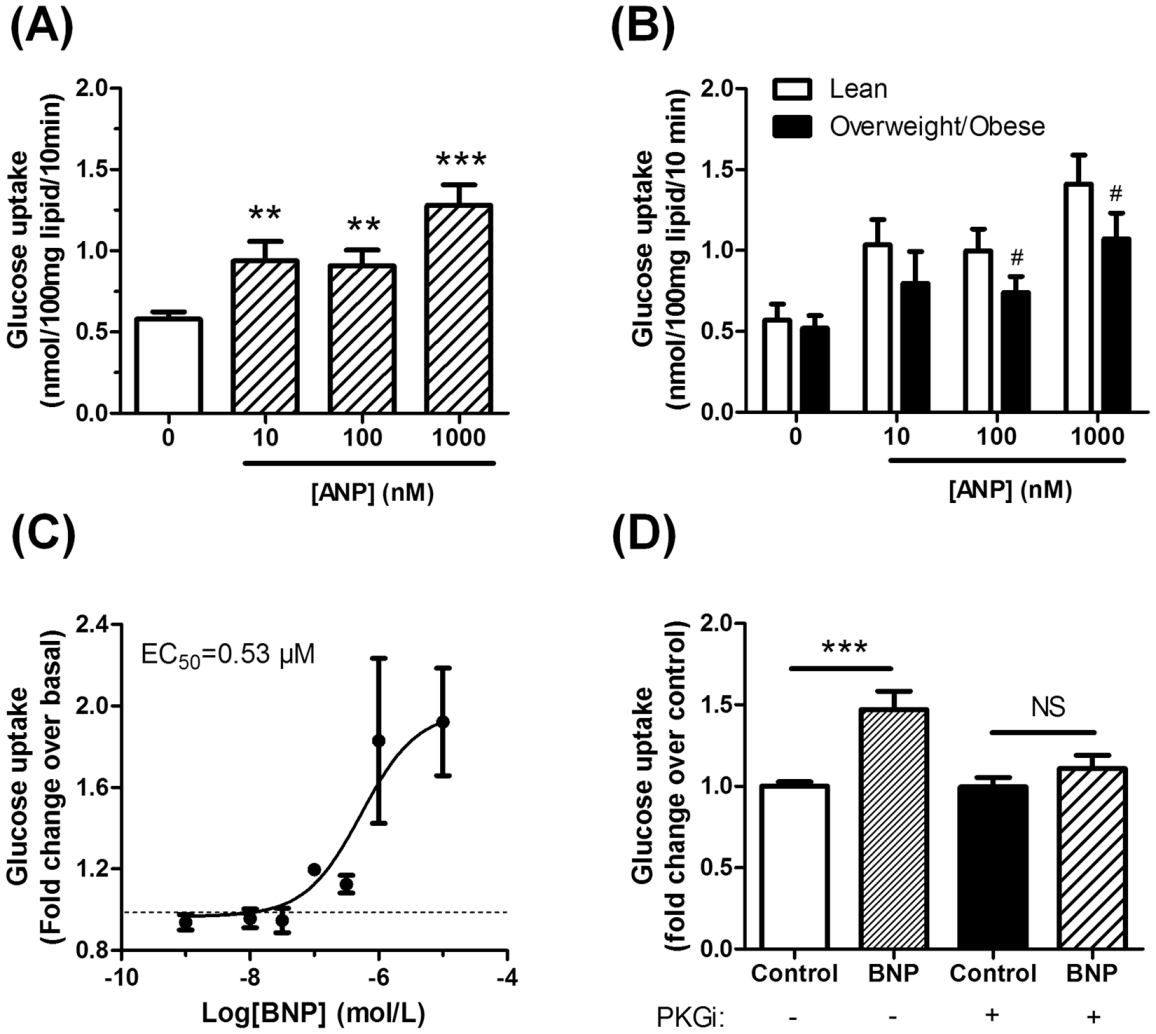
Natriuretic peptides promote glucose uptake in a cGMP-dependent manner in human adipocytes. (**A**) Dose-response effect of ANP on 2-deoxyglucose uptake in human isolated adipocytes (n = 13). (**B**) Dose-response effect of ANP on 2-deoxyglucose uptake in human isolated adipocytes from lean (n = 14) versus overweight/obese subjects (n = 9). ^#^p = 0.06 vs. lean. (**C**) Dose-response effect of BNP on 2-deoxyglucose uptake in differentiated hMADS adipocytes (n = 8). (**D**) BNP (100 nM)-mediated glucose uptake in absence or presence of (Rp)-8-pCPT-cGMPS 100 µM (PKG inhibitor, PKGi) in differentiated hMADS adipocytes (n = 8). *p < 0.05, **p < 0.01, ***p < 0.0001 vs. 0 or control.

We therefore directly assessed the effect of ANP on glucose uptake in human isolated adipocytes obtained from surgical samples. ANP dose-dependently activated basal glucose uptake with a 1.6 fold increase at doses of 10 and 100 nM, and a 2.2 fold increase at the highest dose of 1 µM (Fig. 3A). Of note, ANP promoted glucose uptake at submaximal insulin concentrations of 1 and 10 nM (Supplemental Fig. 3A). Importantly, ANP-mediated glucose uptake was impaired in isolated adipocytes from obese subjects when compared to lean (Fig. 3B). A similar effect was observed for insulin (Supplemental Fig. 3B). To investigate the underlying molecular mechanisms, we next switched to an established human adipocyte cell model system, i.e. human multipotent adipose-derived stem cells (hMADS)^25^. This cell model has been previously used to study lipolysis and the browning process in response to NP^12,26^. These cells express all the components of the NPR signalling pathway evidenced by the fact that *GC-A* mRNA emerged early in the time-course of adipocyte differentiation (day 3) and remained at steadily high levels till the end of the differentiation process (day 13) (Supplemental Fig. 4). *NPRC* and *PRKGI* were expressed at lower levels throughout the time-course of differentiation (Supplemental Fig. 4). Similar to the findings obtained in freshly isolated human adipocytes, ANP (Supplemental Fig. 5A) and BNP (Fig. 3C) induced a dose-dependent increase in glucose uptake that resulted in a 1.5 and 2 fold maximal activation in differentiated hMADS cells. In comparison, the maximal insulin-induced glucose uptake was about 2.5 fold (Supplemental Fig. 5B). ANP and BNP stimulated glucose uptake significantly starting from concentrations of 100 nM and upwards displaying EC_50_ of 0.24 and 0.53 µM, respectively. It is worth mentioning at this stage that no significant effect of the specific NPRC agonist cANP_4-23_ on glucose uptake could be noticed (data not shown). We next used BNP, that is more stable than ANP, for all subsequent experiments. Interestingly, BNP-induced glucose uptake was blunted in presence of (Rp)-8-pCPT-cGMPS 100 µM, a specific pharmacological inhibitor of cGK (PKGi) (Fig. 3D). Collectively, these results indicate that NP promote glucose uptake in a cGMP-dependent manner in human adipocytes.

### Natriuretic peptides activate Akt-signalling in human adipocytes

**Figure 4:**
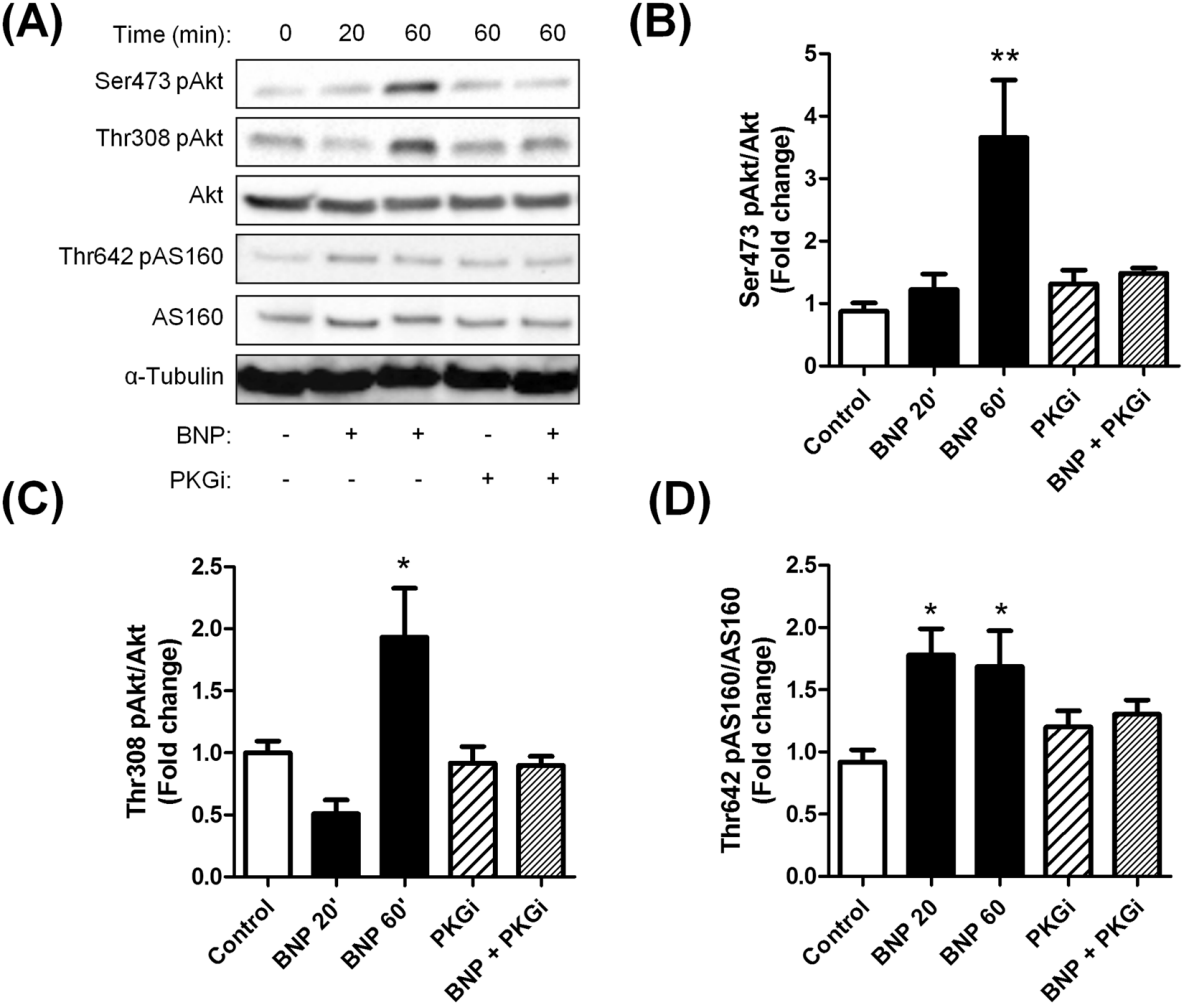
Natriuretic peptides activate Akt-signalling in a cGMP-dependent manner in hMADS adipocytes. Representative blots (**A**) and quantitative bar graphs of Akt Ser473 phosphorylation relative to total Akt (**B**), Akt Thr308 phosphorylation relative to total Akt (**C**), AS160 Thr642 phosphorylation relative to total AS160 (**D**), in response to 20 min and 60 min treatment with BNP 100 nM in absence or presence of (Rp)-8-pCPT-cGMPS 100 µM (PKG inhibitor, PKGi). *p < 0.05, **p < 0.01, ***p < 0.0001 vs. control (n = 6).

Glucose uptake in human adipocyte is mediated by the glucose transporter GLUT4 in response to insulin through activation of the IRS1-PI3K-Akt-signalling pathway^27,28^. Short-term treatment with BNP induced a time-dependent activation of Akt Ser473 phosphorylation, nearing 1.23 fold at 20 min and 3.7 fold at 60 min (p < 0.0001) (Fig. 4A,B). This effect was completely abolished by the cGK inhibitor (Rp)-8-pCPT-cGMPS. Similarly, BNP induced a 2-fold induction of Akt Thr308 phosphorylation that was totally abrogated by cGK inhibition (Fig. 4A–C). BNP-induced Akt activation was further associated with a downstream activation of AS160, a GTPase involved in GLUT4 translocation to the plasma membrane. As for Akt, BNP-mediated phosphorylation of AS160 was totally abrogated by the addition of a pharmacological inhibitor of cGK (Fig. 4A–D). We also confirmed previous findings^12^ showing that BNP treatment induces p38 MAPK (3.2 fold, p < 0.0001) in a cGK-dependent manner (Supplemental Fig. 6).

### Natriuretic peptides activate mTOR-signalling in human adipocytes

**Figure 5:**
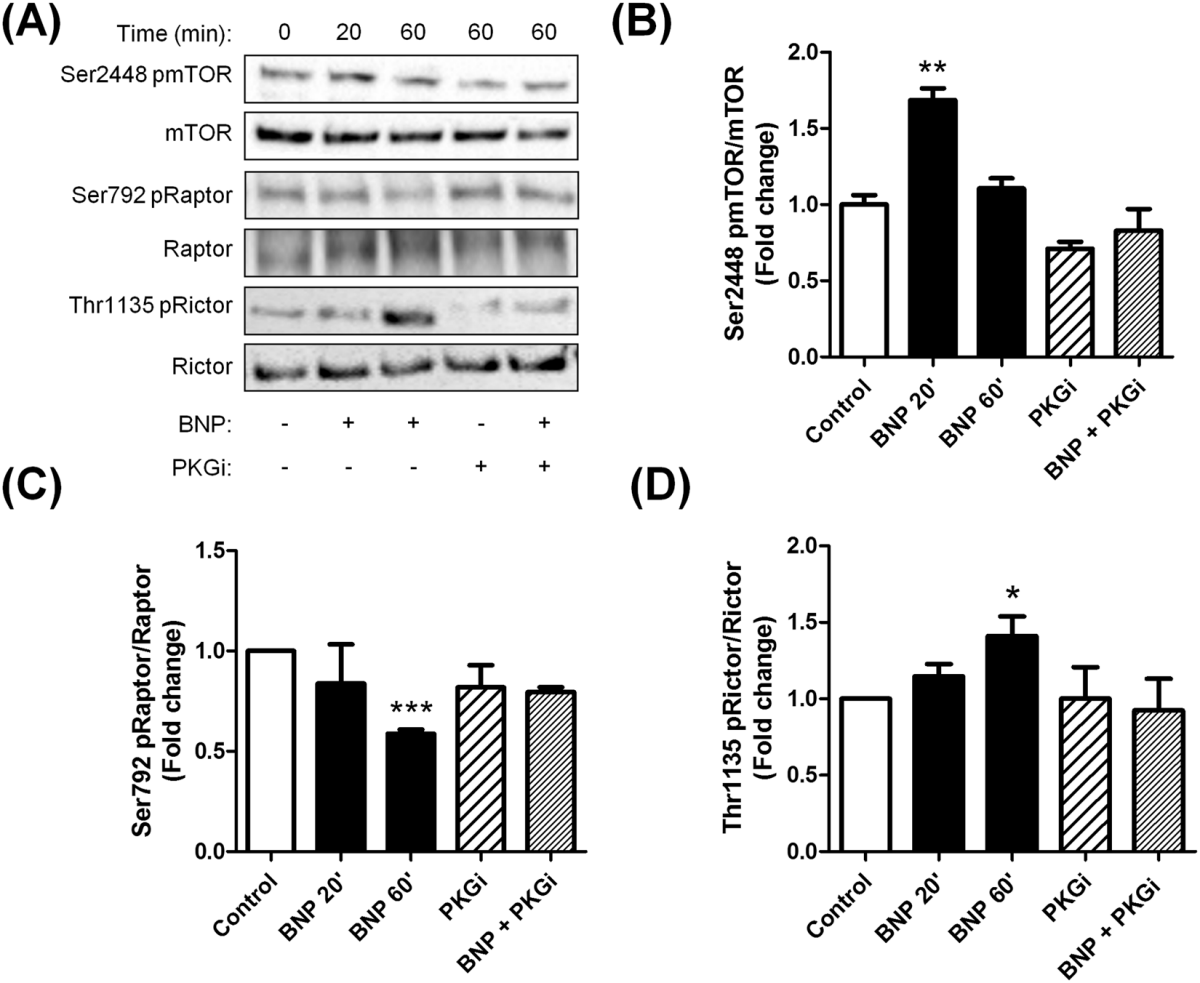
Natriuretic peptides activate mTOR-signalling in a cGMP-dependent manner in hMADS adipocytes. Representative blots (**A**) and quantitative bar graphs of mTOR Ser2448 phosphorylation relative to total mTOR (**B**), Raptor Ser792 phosphorylation relative to total Raptor (**C**), Rictor Thr1135 phosphorylation relative to total Rictor (**D**), in response to 20 min and 60 min treatment with BNP 100 nM in absence or presence of (Rp)-8-pCPT-cGMPS 100 µM (PKG inhibitor, PKGi). *p < 0.05, **p < 0.01, ***p < 0.0001 vs. control (n = 6).

Akt has been shown to be phosphorylated at Ser473 by mTOR complex 2 (mTORC2)^29^. Consistent with this, BNP-mediated phosphorylation of Akt was associated with increased mTOR (Fig. 5A,B), reduced Raptor (Fig. 5A–C), and increased Rictor (Fig. 5A–D) phosphorylation, indicating an activation of the mTORC1 and mTORC2 complexes respectively. Importantly, we further show that pharmacological inhibition of mTOR by rapamycin totally abrogates BNP-mediated glucose uptake (Supplemental Fig. 7A) and Akt Ser473 phosphorylation (Supplemental Fig. 7B).

In summary, our data demonstrate that NP-mediated glucose uptake requires mTORC1/2 to induce Akt signalling in human adipocytes.

### Natriuretic peptides enhance glucose metabolism in human adipocytes

**Figure 6:**
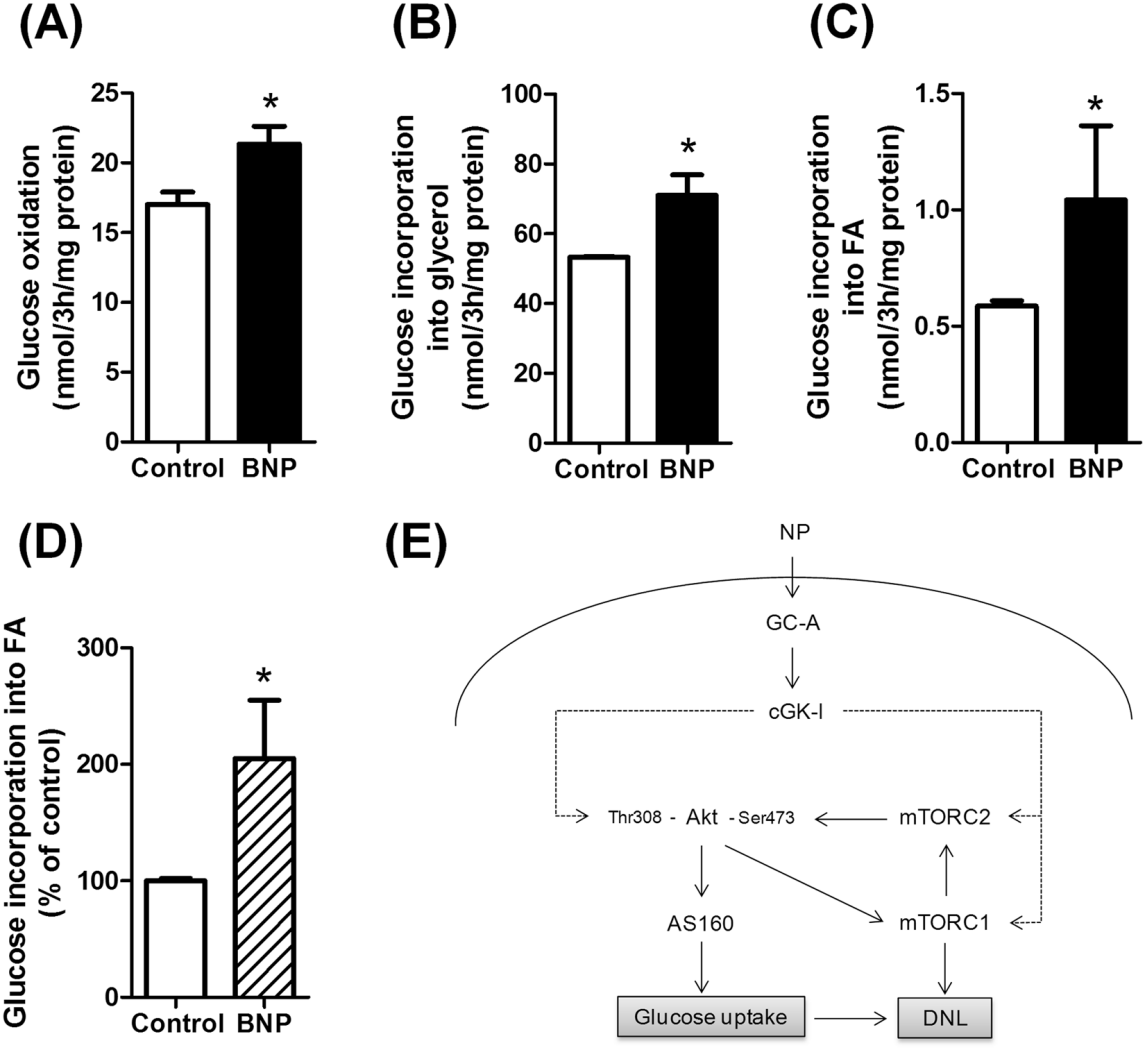
Natriuretic peptides enhance glucose metabolism in human adipocytes. Effect of acute BNP 100 nM treatment on glucose oxidation (**A**), glucose incorporation into glycerol (**B**), and glucose incorporation into fatty acids (FA) (**C**) in hMADS adipocytes. (**D**) Effect of 100 nM BNP on glucose incorporation into FA in human isolated adipocytes. *p < 0.05 vs. control (n = 4–7). (**E**) Schematic model of NP-mediated glucose uptake in human adipocytes. Natriuretic peptides (NP) bind to a transmembrane receptor bearing a guanylyl cyclase activity called GC-A. Binding of NP to GC-A induces the production of cGMP and activation of cGK-I which activate Akt and mTORC2. Activation of mTORC2 phosphorylates Akt at Ser473 and enhances downstream signalling to the Rab-GTPase AS160 which promotes membrane GLUT4 translocation and glucose uptake. NP therefore promote glucose uptake and enhance glucose incorporation into glycerol and FA pools through *de novo* lipogenesis.

We finally investigated the fate of glucose taken up by adipocytes in response to NP. Acute BNP treatment (3 h) induced both glucose oxidation (+19% versus control, p < 0.05) (Fig. 6A), and glucose incorporation into glycerol (+33% versus control, p < 0.05) (Fig. 6B) or fatty acids (+78% versus control, p < 0.05) (Fig. 6C) in hMADS adipocytes. No significant change in mRNA levels of prototypical *de novo* lipogenic genes such as *MLXIPL* (ChREBP), *ACC1*, *FASN*, and *ELOVL6* was observed in response to 6 h treatment with ANP or BNP (Supplemental Fig. 8). No significant change in GLUT1 and GLUT4 mRNA levels were observed as well (data not shown). BNP also induced about 2-fold the incorporation of glucose into fatty acids in human isolated adipocytes (Fig. 6D).

## Discussion

Despite the robust inverse link between plasma NP levels, obesity and the incidence of T2D, no study has so far provided data showing a direct mechanistic link between adipose tissue NP signalling and glucose homeostasis. Herein, we provide some evidence that NP receptor expression in adipose tissue is tightly related to blood glucose control and insulin sensitivity in different European populations. We further demonstrate a novel biological role of NP in human adipocytes where they directly promote glucose uptake and *de novo* lipogenesis in fat cells through a cGMP-dependent pathway (Fig. 6E). Overall, our data suggest that NP signalling in human adipocytes is an important determinant of insulin sensitivity that is altered in obesity and T2D.

Previous studies have established that adipose tissue is a key target organ of NP^12,21,30,31^. In this study, we took advantage of a large adipose tissue collection from cohort 1 to investigate NPR gene expression in the context of obesity and T2D. Due to the large sample size, we observed a very strong negative correlation between adipose *GC-A* gene expression and insulin resistance, suggesting that low adipose GC-A expression relates to low insulin sensitivity. In contrast, adipose *NPRC* mRNA levels were not associated with insulin resistance. Recent studies indicate that adipose glucose transporter-4 (GLUT4) and carbohydrate-responsive element binding protein (ChREBP, encoded by *MLXIPL*) are major determinants of adipocyte and whole-body insulin sensitivity^23,24^. In the current study, we found very robust relationships between adipose *GC-A*, HOMA-IR, *GLUT4* and *MLXIPL* mRNA levels in two independent cohorts. These relationships remained significant also after statistical adjustment for BMI in cohort 2, indicating that adipose GC-A behaves as a determinant of whole-body insulin sensitivity independently of body weight. In addition, we observed major differences in adipose tissue NPR expression in relation to degree of obesity and diabetic status. Due to paucity of tissue sample it was not possible to also measure protein expression. However previous studies in human adipose tissue suggest a close relationship. Thus, in agreement with recent studies^7,32,33^ reporting on the mRNA and protein levels of GC-A and NPRC in obese and non-obese subjects and mice, *GC-A* expression was negatively and *NPRC* positively associated with BMI. Moreover, *GC-A* mRNA levels were lower in individuals with prediabetes and T2D compared with NGT subjects, an observation that fits well with previously reported animal studies in high-fat diet-fed and db/db mice^32,34^. Our current data are also in line with a recent study from Kovacova *et al*. demonstrating a down-regulation of the GC-A-to-NPRC ratio in obese versus lean individuals as well as a strong link with whole-body insulin sensitivity^31^.

In light of the tight relationship observed between adipose GC-A, *de novo* lipogenesis and the lipogenic genes *GLUT4* and *MLXIPL*, we next studied the effect of NP on glucose uptake in human adipocytes. We could first show that ANP dose-dependently activated glucose uptake in human isolated adipocytes. Although both the cGMP and the cAMP pathways promote lipolysis and browning of white adipocytes^6^, they display contrasting effects with respect to glucose uptake which is inhibited by activating the cAMP-signalling pathway^35,36^. Thus catecholamines inhibit glucose uptake by inducing lipolysis and facilitating the dissociation of the mTORC1/2 complex^36^. In the current study, we provide evidence of a NP-mediated glucose uptake in adipocytes, which is independent of insulin and requires downstream cGMP-signalling since pharmacological inhibition of cGK totally abrogates NP-mediated glucose uptake. Although cGMP has been shown to mediate glucose uptake in skeletal muscle^37^, this is the first study reporting that activation of cGMP-signalling by NP promotes glucose uptake in human adipocytes.

Insulin promotes glucose uptake in skeletal muscle cells and adipocytes through activation of the phosphadityl-inositol-3-kinase/Akt pathway leading to GLUT4 translocation to the plasma membrane^27,28^. We here demonstrate that BNP treatment in hMADS adipocytes induces Akt phosphorylation at both Thr308 and Ser473 residues. Again this effect appears to be mediated by cGMP since pharmacological blockade of cGK completely abrogated BNP-mediated Akt phosphorylation. Since Akt phosphorylation at Ser473 requires mTORC2 activation^29^, we further show that BNP treatment was able to induce mTORC2 through phosphorylation of mTOR and Rictor. We also show that NP signalling induces mTORC1 by modulating both mTOR and Raptor. It is possible that cGK-I directly phosphorylates mTOR and Raptor, since cAMP-dependent protein kinase was recently shown to do so^38^. We could next demonstrate that pharmacological inhibition of mTORC1/2 by rapamycin totally suppresses NP-mediated Akt phosphorylation at Ser473 and glucose uptake. Altogether, this suggests that cGK-I may indirectly modulate Akt signalling through mTORC1/2. Our data are overall consistent with previous work showing the critical role of mTORC1/2 in the control of glucose metabolism in adipocytes^36,39,40^. Our data are also in line with recent data from the Collins’ group showing that enhanced NP signalling by deleting *Npr3* specifically in mouse adipose tissue is associated with higher phosphorylation of Akt at Ser473 and Thr308^41^. Interestingly, BNP-mediated Akt activation was accompanied by an elevated phosphorylation of the Rab GTPase-activating protein AS160 (also termed TBC1D4), which coordinates GLUT4 translocation to the plasma membrane in adipocytes and myocytes^42,43^.

Considering the tight relationship observed between adipose *GC-A* and *MLXIPL* mRNA levels, we examined the fate of the glucose taken up by adipocytes in response to NP. We could demonstrate that about one quarter of the glucose taken up by the adipocyte was directed toward glucose oxidation while the remaining three quarters were incorporated into the glycerol backbone for triglyceride synthesis. Of interest, a quantitatively minor fraction of glucose (less than 1%) served for *de novo* production of fatty acids, i.e. *de novo* lipogenesis. Thus activation of NP signalling in adipocytes enhances *de novo* lipogenesis. No significant changes in prototypical genes of *de novo* lipogenesis were observed after NP treatment indicating that NP-mediated de *novo* lipogenesis likely results from NP-mediated glucose uptake independently of the transcriptional activity of the glucose-regulated transcription factor ChREBP.

In light of the potent lipolytic effect of NP previously observed in human adipocytes^9^, it may seem paradoxical at first glance that NP can also trigger glucose uptake. Although speculative, this could reflect some sort of futile cycle by which NP firstly enhance glucose uptake and *de novo* lipogenesis, and then promote triglyceride breakdown through lipolysis and fatty acid oxidation. The effect of NP on glucose uptake is also consistent with their browning effect in white adipocytes as glucose is an important substrate for brown adipocytes^12^, and this futile cycle lipogenesis/lipolysis could provide an additional energy dissipating process in beige/brown adipocytes^44^. Indeed, futile cycling between *de novo* lipogenesis and fatty acid oxidation has been shown to be induced in adipose tissues during cold exposure or stimulation with a β-adrenergic agonist as recently discussed^45^. High rates of *de novo* lipogenesis are observed in brown adipocytes during cold exposure supplying approximately 30% of oxidized FA required for heat production^46–48^. Such a dual effect on glucose uptake and lipolysis has also been observed in rat adipocytes treated with β-sitosterol^49^.

We obviously acknowledge a number of limitations in the present work: 1) We were not able to measure GC-A and NPRC protein expression, to corroborate mRNA data, as no sample was prepared and/or biopsy material left for this type of analysis in both clinical cohorts. 2) We did not fully elucidate the precise signalling pathway by which NP promote glucose uptake. However we can also emphasize the strengths of the current study: 1) Gene expression data and associations with surrogates of insulin sensitivity were observed in a large sample size from two independent cohorts. 2) The effect of NP on glucose uptake was consistently observed in isolated human mature adipocytes and cultured hMADS adipocytes. 3) Our data are in line with a recent study indicating that enhancing NP signalling specifically in adipose tissue improves insulin signalling in white and brown fat, systemic insulin sensitivity and glucose tolerance^41^.

In summary, our data suggest an important role of NP in regulating adipocyte glucose metabolism and insulin sensitivity in humans. This occurs through GC-A in a cGMP-dependent manner in parallel of the insulin pathway. Our clinical data also argue that adipose GC-A expression is tightly associated with whole-body insulin sensitivity. Future studies should investigate the causal link between the NP/GC-A system and T2D before considering GC-A activation as a potential target to improve blood glucose control and insulin sensitivity.

## Methods

Details for cultures of hMADS adipocytes can be found in Supplementary Material.

### Clinical studies and human subject**s**

#### Cohort 1

The samples investigated in this paper were collected from 2006 to 2007 during the DiOGenes study, a pan-European randomized trial, which was approved by the ethics committees of each of the 8 European centers participating to the program (NCT00390637). The DiOGenes project investigated the effects of diets with different content of protein and glycemic index on weight-loss maintenance and metabolic and cardiovascular risk factors after an 8-week calorie restriction phase, in obese/overweight individuals. Written informed consent was obtained from each patient according to the local ethics committee of the participating countries as previously described^50^.

Healthy overweight (body mass index (BMI) ≥ 27 kg/m^2^) individuals, aged <65 years were eligible for the study. Exclusion criteria were BMI >45 kg/m^2^, liver or kidney diseases, cardiovascular diseases, diabetes mellitus type 1, special diets/eating disorders, systemic infections/chronic diseases, cancer within the last 10 years, weight change >3 kg within the previous 3 months, and other clinical disorders or use of prescription medication that might interfere with the outcome of the study.

A detailed description of inclusion and exclusion criteria has been published previously^51^. BMI was calculated by dividing weight in kilograms by the square of height in meters. A detailed description of the DiOGenes intervention trial and main outcomes can be found in the core publication^50^. Briefly, among 1209 individuals screened, 932 entered a baseline clinical investigation day including anthropometric measures (height, weight, waist circumference, body composition), blood pressure measurements, fasting blood sampling, and subcutaneous adipose tissue biopsies were performed (at baseline and at the end of each phase). All procedures were standardized between the 8 study centers across Europe.

#### Cohort 2

Cohort 2 comprised 30 obese (BMI >30 kg/m^2^) otherwise healthy and 26 non-obese (BMI <30 kg/m^2^) healthy women that have been described in detail previously^52^. All were pre-menopausal and free of continuous medication. They were investigated in the morning after an overnight fast in the midst of their menstrual cycle. A venous blood sample was obtained for measurements of glucose and insulin and the values were used to calculate HOMA-IR^53^. An abdominal subcutaneous adipose tissue biopsy was obtained by needle aspiration as described^54^.

### Gene microarrays

From adipose tissue biopsy total RNA in Cohort 2, biotinylated complementary RNA was analyzed using the GeneChip Human Gene 1.0 ST Array (Affymetrix Inc., Santa Clara, CA). Slides were washed, stained, scanned and analyzed using standardized protocols (Affymetrix Inc.) as described previously^52^. Data are deposited at the National Center for Biotechnology Information Gene Expression Omnibus (GEO; http://ncbi.nim.nih.gov/geo) under the accession number GSE25402.

### Microfluidic card

Total RNA was extracted from adipose tissue biopsies and RT-qPCR was performed using the FluidigmBioMark System as described in^55^. Briefly, cDNA was prepared from 500 ng of total RNA and diluted in water to 5 ng/µL (RNA equivalent). The reverse transcription step was checked using *HPRT* expression level using StepOnePlus (Applied Biosystems). A multiplexed preamplification process was performed on every 1.25 µL cDNA using 14 cycle cDNA preamplification step (95 °C for 15 sec and 60 °C 4 min) and Taqman PreAmp Master Mix (Applied Biosystems) in a standard PCR thermocycler. Preamplified cDNA was diluted 1:5 in 10 mM Tris, 1 mM EDTA (TE). Diluted cDNA (2.25 µL) was added to 2.5 µL Taqman Universal PCR Master Mix (Applied Biosystems) and 0.25 µL GE Sample Loading Reagent (Fluidigm). In a separate tube, 3.5 µL of Taqman Assay was added to 3.5 µL Sample Loading Reagent. Five µL cDNA samples were loaded into the sample inlet wells, and 5 µL assay samples were loaded into assay detector inlets. For each plate, 1 well was loaded with H_2_0 as control for contamination. The chip was primed and placed into the NanoFlex Integrated fluidic circuit controller where 8 nL of cDNA and 1 nL of Assay were mixed. Real time PCR was run on the BioMark System (Fluidigm). Raw data obtained from the system’s software using the default global threshold setting (BioMark Real-time PCR Analysis V2.1.1, Fluidigm) were checked using the graphical representation of the plate layout. PUM1, was found as the most stable gene using the geNorm algorithm^56^, then raw Ct values were transformed to relative gene expression using the 2^(ΔCt)^ method using PUM1 mRNA level as reference.

### Real-time qRT-PCR

Total RNA from cultured hMADS cells was isolated in RNeasy Lysis Buffer ± mercaptoethanol reagent (Qiagen GmbH, Hilden, Germany). The quantity of the RNA was determined on a Nanodrop ND-1000 (Thermo Scientific, Rockford, IL, USA). Reverse transcriptase PCR was performed on a GeneAmp PCR System 9700 using the Multiscribe Reverse Transcriptase method (Applied Biosystems, Foster City, CA). Real-time quantitative PCR (qPCR) was performed to determine cDNA content. All primers were bought from Applied Biosystems. Primers used were: 18S (Taqman assay ID: Hs99999901_s1), ACC1 (Hs00167385_m1), FAS (Hs00188012_m1), ChREBP (Hs00975714_m1). ELOVL6, SYBR green primers, forward: CCATCCAATGGATGCAGGAAAAC; reverse: CCAGAGCACTAATGGCTTCCTC were purchased at Eurogentec. qPCR was then performed on a StepOnePLus real-time PCR system (Applied Biosystems). For each primer, a standard curve was made prior to mRNA quantification to assess the optimal total cDNA quantity. All expression data were normalized by the 2^(ΔCt)^ method using 18S as internal control^55,57^.

### Glucose uptake assay in isolated mature adipocytes

Fat cell suspensions were incubated without (baseline) or with insulin or ANP for 45 min at 37 °C, pH 7.5 in 400 μL final volume of incubation medium (see supplemental methods). Then, an isotopic dilution of 2-deoxy-D-[^3^H]glucose (2-DG) was added to reach 50 nmol and 1,000,000 dpm in each assay tube that contained an average amount of fat cells equivalent to 19 ± 1 mg lipids, and that was further incubated for an additional 10-min period as previously described^58^. After stopping by addition of 100 μM cytochalasin B, cell suspension aliquots were centrifuged through diisononyl-phthalate layer to separate the buoyant adipocytes from the medium allowing counting the intracellular radioactive 2-DG, as an index of glucose uptake, which was expressed as nmol 2-DG internalized/100 mg cellular lipids/10 min, as previously reported^59^.

### Glucose uptake assay in hMADS adipocytes

The day before the assay, insulin was removed from culture medium. After two washes with PBS, cells were incubated 50 min at 37 °C without or with various concentrations of BNP (10^−11^ to 10^−5^ M), ANP (10^−9^ to 10^−5^ M) or insulin. Then, 125 µM 2-deoxy-D-glucose and 0.4 µCi 2-DG per well were added for 10 min incubation. Culture plates were put on ice and rinsed with 10 mM glucose in ice-cold PBS and then with ice-cold PBS. Cells were scraped in 0.05 N NaOH and 2-deoxy-D-glucose uptake was measured by liquid scintillation counting of cell lysate. Data are expressed as nanomoles per minute and were normalized per mg of protein^60^.

### Determination of glucose oxidation

The day before the assay, insulin was removed from culture medium. Cells were preincubated with a glucose- and serum-free medium for 90 min then exposed to DMEM low glucose (5.5 mM) supplemented with D-[U-^14^C]glucose (1 µCi/mL; PerkinElmer, Boston, MA) in the presence or absence of 100 nM BNP for 3 h. Following incubation, glucose oxidation rate was determined by measuring [^14^C]CO_2_ by liquid scintillation counting as previously described^57^. Briefly, assayed medium is transferred into a custom–made Teflon 48-well trapping plate. The plate is clamped and sealed, and perchloric acid is injected through the perforations in the lid into the medium, which drives CO2 through the tunnel into an adjacent well, where it is trapped in 1N NaOH. Following trapping, the total volume of NaOH is transferred into scintillation vials, and radioactivity is measured on a multipurpose scintillation counter (LS 6500; Beckman Coulter). All assays are performed in triplicates, and data are normalized to total protein content.

### Determination of glucose incorporation into glycerol and fatty acids

To determine glucose carbon incorporation into fatty acid and glycerol, after the glucose oxidation as described above, adipocytes were collected in SDS 0.1% and total lipids were extracted in chloroform/methanol (2:1) according to Folch *et al*.^61^. After centrifugation, the organic phase was dried under nitrogen and hydrolyzed in 1 mL 0.25 N NaOH in chloroform/methanol (1:1) for 1 h at 37 °C. The solution was neutralized with 500 mL 0.5 N HCl in methanol. FA and glycerol were separated by adding 1.7 mL chloroform, 860 mL water, and 1 mL chloroform/methanol (2:1). Incorporation of ^14^C into glycerol and FA was measured by liquid scintillation counting of upper and lower phases, respectively. Specific activity was counted and used to determine the quantity of incorporated glucose equivalent. Data were normalized to total cell protein content.

### Western blot

Differentiated hMADS cell lysates were extracted, transferred onto nitrocellulose membranes and blotted with the following primary antibodies (all from Cell Signalling Technology Inc., Beverly, MA): phospho-Akt Ser473 (#4060), phospho-Akt Thr308 (#2965), Akt (#4691), phospho-IRS1 Tyr612 (#44816G), IRS1 (#3407), phospho-AS160 Thr642 (#4288), AS160 (#2670), phospho-p38 MAPK Thr180/Tyr182 (#9211), and p38 MAPK (#9212), phospho-Raptor Ser792 (#2083), Raptor (#2280), phospho-Rictor Thr1135 (#3606), Rictor (#2140), phospho-mTOR Ser2448 (#2971), mTOR (#2972). Immunoreactive proteins were detected by enhanced chemiluminescence reagent (SuperSignal West Dura or SuperSignal West Femto; Thermo Scientific), visualized using the ChemiDoc MP Imaging System and data analyzed using the Image Lab 4.1 version software (Bio-Rad Laboratories, Hercules, USA). α-tubulin (Sigma-Aldrich) was used as internal control.

### Statistical analyses

All statistical analyses were performed using GraphPad Prism 5.0 for Windows (GraphPad Software Inc., San Diego, CA). Normal distribution and homogeneity of variance of the data were tested using Shapiro-Wilk and F tests, respectively. One-way ANOVA followed by Tukey’s post-hoc tests and Student’s *t-*tests were performed to determine differences between groups, interventions and treatments. Two-way ANOVA followed by Bonferonni’s post hoc tests were applied when appropriate. Linear regression was performed after log transformation of nonparametric data. The false discovery rate for multiple testing was controlled by the Benjamini-Hochberg procedure with p_adj_. values ≤ 0.05 as threshold. All values in Figures and Tables are presented as mean ± SEM. Statistical significance was set at p < 0.05.

## Electronic supplementary material

Supplementary Information

## References

[1] Gardner DG, Chen S, Glenn DJ, Grigsby CL (2007). Molecular biology of the natriuretic peptide system: implications for physiology and hypertension. Hypertension.

[2] Garg R, Pandey KN (2005). Regulation of guanylyl cyclase/natriuretic peptide receptor-A gene expression. Peptides.

[3] Kuhn M (2005). Cardiac and intestinal natriuretic peptides: insights from genetically modified mice. Peptides.

[4] Kuhn M (2016). Molecular Physiology of Membrane Guanylyl Cyclase Receptors. Physiological reviews.

[5] Moro C, Lafontan M (2013). Natriuretic peptides and cGMP signaling control of energy homeostasis. Am J Physiol Heart Circ Physiol.

[6] Collins S, Bordicchia M (2013). Heart hormones fueling a fire in fat. Adipocyte.

[7] Coue M (2015). Defective Natriuretic Peptide Receptor Signaling in Skeletal Muscle Links Obesity to Type 2 Diabetes. Diabetes.

[8] Moro C (2016). Targeting cardiac natriuretic peptides in the therapy of diabetes and obesity. Expert opinion on therapeutic targets.

[9] Sengenes C, Berlan M, De Glisezinski I, Lafontan M, Galitzky J (2000). Natriuretic peptides: a new lipolytic pathway in human adipocytes. FASEB journal: official publication of the Federation of American Societies for Experimental Biology.

[10] Sengenes C (2003). Involvement of a cGMP-dependent pathway in the natriuretic peptide-mediated hormone-sensitive lipase phosphorylation in human adipocytes. The Journal of biological chemistry.

[11] Birkenfeld AL (2012). Atrial natriuretic peptide and adiponectin interactions in man. PLoS One.

[12] Bordicchia M (2012). Cardiac natriuretic peptides act via p38 MAPK to induce the brown fat thermogenic program in mouse and human adipocytes. The Journal of clinical investigation.

[13] Wang TJ (2004). Impact of obesity on plasma natriuretic peptide levels. Circulation.

[14] Mehra MR (2004). Obesity and suppressed B-type natriuretic peptide levels in heart failure. J Am Coll Cardiol.

[15] Taylor JA, Christenson RH, Rao K, Jorge M, Gottlieb SS (2006). B-type natriuretic peptide and N-terminal pro B-type natriuretic peptide are depressed in obesity despite higher left ventricular end diastolic pressures. Am Heart J.

[16] Khan AM (2011). Cardiac natriuretic peptides, obesity, and insulin resistance: evidence from two community-based studies. J Clin Endocrinol Metab.

[17] Lazo M (2013). NH2-terminal pro-brain natriuretic peptide and risk of diabetes. Diabetes.

[18] Magnusson M (2012). Low plasma level of atrial natriuretic peptide predicts development of diabetes: the prospective Malmo Diet and Cancer study. J Clin Endocrinol Metab.

[19] Walford GA (2014). Circulating natriuretic peptide concentrations reflect changes in insulin sensitivity over time in the Diabetes Prevention Program. Diabetologia.

[20] Sarzani R (1995). Fasting inhibits natriuretic peptides clearance receptor expression in rat adipose tissue. J Hypertens.

[21] Sarzani R, Dessi-Fulgheri P, Paci VM, Espinosa E, Rappelli A (1996). Expression of natriuretic peptide receptors in human adipose and other tissues. J Endocrinol Invest.

[22] Dessi-Fulgheri P (1999). Low calorie diet enhances renal, hemodynamic, and humoral effects of exogenous atrial natriuretic peptide in obese hypertensives. Hypertension.

[23] Eissing L (2013). *De novo* lipogenesis in human fat and liver is linked to ChREBP-beta and metabolic health. Nature communications.

[24] Herman MA (2012). A novel ChREBP isoform in adipose tissue regulates systemic glucose metabolism. Nature.

[25] Rodriguez AM (2004). Adipocyte differentiation of multipotent cells established from human adipose tissue. Biochemical and biophysical research communications.

[26] Bezaire V (2009). Contribution of adipose triglyceride lipase and hormone-sensitive lipase to lipolysis in hMADS adipocytes. The Journal of biological chemistry.

[27] Klip A, Sun Y, Chiu TT, Foley KP (2014). Signal transduction meets vesicle traffic: the software and hardware of GLUT4 translocation. American journal of physiology. Cell physiology.

[28] Graham TE, Kahn BB (2007). Tissue-specific alterations of glucose transport and molecular mechanisms of intertissue communication in obesity and type 2 diabetes. Hormone and metabolic research = Hormon- und Stoffwechselforschung = Hormones et metabolisme.

[29] Sarbassov DD, Guertin DA, Ali SM, Sabatini DM (2005). Phosphorylation and regulation of Akt/PKB by the rictor-mTOR complex. Science.

[30] Moro C (2007). Atrial natriuretic peptide inhibits the production of adipokines and cytokines linked to inflammation and insulin resistance in human subcutaneous adipose tissue. Diabetologia.

[31] Kovacova Z (2016). Adipose tissue natriuretic peptide receptor expression is related to insulin sensitivity in. obesity and diabetes. Obesity (Silver Spring).

[32] Miyashita K (2009). Natriuretic peptides/cGMP/cGMP-dependent protein kinase cascades promote muscle mitochondrial biogenesis and prevent obesity. Diabetes.

[33] Ryden, M. *et al*. Impaired atrial natriuretic peptide-mediated lipolysis in obesity. *Int J Obes (Lond)* (2015). 10.1038/ijo.2015.222 26499437

[34] Plante E (2014). Treatment with brain natriuretic peptide prevents the development of cardiac dysfunction in obese diabetic db/db mice. Diabetologia.

[35] Eriksson JW, Wesslau C, Smith U (1994). The cGMP-inhibitable phosphodiesterase modulates glucose transport activation by insulin. Biochimica et biophysica acta.

[36] Mullins GR (2014). Catecholamine-induced lipolysis causes mTOR complex dissociation and inhibits glucose uptake in adipocytes. Proceedings of the National Academy of Sciences of the United States of America.

[37] Deshmukh AS (2010). Nitric oxide increases cyclic GMP levels, AMP-activated protein kinase (AMPK)alpha1-specific activity and glucose transport in human skeletal muscle. Diabetologia.

[38] Liu D (2016). Activation of mTORC1 is essential for beta-adrenergic stimulation of adipose browning. The Journal of clinical investigation.

[39] Tang Y (2016). Adipose tissue mTORC2 regulates ChREBP-driven *de novo* lipogenesis and hepatic glucose metabolism. Nature communications.

[40] Veilleux A, Houde VP, Bellmann K, Marette A (2010). Chronic inhibition of the mTORC1/S6K1 pathway increases insulin-induced PI3K activity but inhibits Akt2 and glucose transport stimulation in 3T3-L1 adipocytes. Mol Endocrinol.

[41] Wu, W. *et al*. Enhancing natriuretic peptide signaling in adipose tissue, but not in muscle, protects against diet-induced obesity and insulin resistance. *Science signaling* **10** (2017). 10.1126/scisignal.aam6870 PMC7418652 28743802

[42] Tan SX (2012). The Rab GTPase-activating protein TBC1D4/AS160 contains an atypical phosphotyrosine-binding domain that interacts with plasma membrane phospholipids to facilitate GLUT4 trafficking in adipocytes. Molecular and cellular biology.

[43] Lansey MN, Walker NN, Hargett SR, Stevens JR, Keller SR (2012). Deletion of Rab GAP AS160 modifies glucose uptake and GLUT4 translocation in primary skeletal muscles and adipocytes and impairs glucose homeostasis. American journal of physiology. Endocrinology and metabolism.

[44] Kajimura S, Spiegelman BM, Seale P (2015). Brown and Beige Fat: Physiological Roles beyond Heat Generation. Cell metabolism.

[45] Solinas G, Boren J, Dulloo AG (2015). *De novo* lipogenesis in metabolic homeostasis: More friend than foe?. Molecular metabolism.

[46] Trayhurn P (1981). Fatty acid synthesis in mouse brown adipose tissue. The influence of environmental temperature on the proportion of whole-body fatty acid synthesis in brown adipose tissue and the liver. Biochimica et biophysica acta.

[47] Yu XX, Lewin DA, Forrest W, Adams SH (2002). Cold elicits the simultaneous induction of fatty acid synthesis and beta-oxidation in murine brown adipose tissue: prediction from differential gene expression and confirmation *in vivo*. FASEB journal: official publication of the Federation of American Societies for Experimental Biology.

[48] de Jesus LA (2001). The type 2 iodothyronine deiodinase is essential for adaptive thermogenesis in brown adipose tissue. The Journal of clinical investigation.

[49] Chai JW, Lim SL, Kanthimathi MS, Kuppusamy UR (2011). Gene regulation in beta-sitosterol-mediated stimulation of adipogenesis, glucose uptake, and lipid mobilization in rat primary adipocytes. Genes & nutrition.

[50] Larsen TM (2010). Diets with high or low protein content and glycemic index for weight-loss maintenance. The New England journal of medicine.

[51] Larsen TM (2010). The Diet, Obesity and Genes (Diogenes) Dietary Study in eight European countries - a comprehensive design for long-term intervention. Obesity reviews: an official journal of the International Association for the Study of Obesity.

[52] Arner E (2012). Adipose tissue microRNAs as regulators of CCL2 production in human obesity. Diabetes.

[53] Matthews DR (1985). Homeostasis model assessment: insulin resistance and beta-cell function from fasting plasma glucose and insulin concentrations in man. Diabetologia.

[54] Kolaczynski JW (1994). A new technique for biopsy of human abdominal fat under local anaesthesia with Lidocaine. International journal of obesity and related metabolic disorders: journal of the International Association for the Study of Obesity.

[55] Viguerie N (2012). Determinants of human adipose tissue gene expression: impact of diet, sex, metabolic status, and cis genetic regulation. PLoS genetics.

[56] Vandesompele J (2002). Accurate normalization of real-time quantitative RT-PCR data by geometric averaging of multiple internal control genes. Genome biology.

[57] Badin PM (2012). Regulation of skeletal muscle lipolysis and oxidative metabolism by the co-lipase CGI-58. Journal of lipid research.

[58] Gomez-Zorita S, Treguer K, Mercader J, Carpene C (2013). Resveratrol directly affects *in vitro* lipolysis and glucose transport in human fat cells. Journal of physiology and biochemistry.

[59] Wanecq E, Prevot D, Carpene C (2009). Lack of direct insulin-like action of visfatin/Nampt/PBEF1 in human adipocytes. Journal of physiology and biochemistry.

[60] Girousse A (2013). Partial inhibition of adipose tissue lipolysis improves glucose metabolism and insulin sensitivity without alteration of fat mass. PLoS biology.

[61] Folch J, Lees M, Sloane Stanley GH (1957). A simple method for the isolation and purification of total lipides from animal tissues. The Journal of biological chemistry.

