# Supplementary material for: Natriuretic peptides promote glucose uptake in a cGMP-dependent manner in human adipocytes
Source: Sci Rep. 2018 Jan 18;8:1097. doi: 10.1038/s41598-018-19619-0 (PMC5773662; doi:10.1038/s41598-018-19619-0)

SUPPLEMENTARY INFORMATION

Natriuretic peptides promote glucose uptake in a cGMP-dependent manner in human adipocytes

Marine Coué[1,2], Valentin Barquissau[1,2], Pauline Morigny[1,2], Katie Louche[1,2], Corinne Lefort[1,2], Aline Mairal[1,2], Christian Carpéné[2,3], Nathalie Viguerie[1,2], Peter Arner[4], Dominique Langin[1,2,5], Mikael Rydén[4], and Cedric Moro[1,2]

[1]INSERM, UMR1048, Institute of Metabolic and Cardiovascular Diseases, Obesity Research Laboratory, Toulouse, France
[2]University of Toulouse, UMR1048, Paul Sabatier University, France
[3]INSERM, UMR1048, Team 1, Institute of Metabolic and Cardiovascular Diseases, Toulouse, France
[4]Department of Medicine-H7, Karolinska Institutet, Karolinska University Hospital, Stockholm, Sweden
[5]Toulouse University Hospitals, Department of Clinical Biochemistry, Toulouse, France

- Supplementary Methods
- Supplementary Figure Legend
- Supplementary Figures: 8
- Supplementary References

Supplementary Methods

Preparation of human isolated adipocytes

Samples of subcutaneous abdominal adipose tissue were obtained from a total of a total of 32 premenopausal women (mean age 38 years; body mass index range: 20.4 – 39.0 kg/m^2) undergoing reconstructive surgery at Rangueil hospital, Toulouse (France) under the agreement of INSERM guidelines and ethics committee. After removal, pieces of adipose tissue were placed in cooled, sterile plastic box and immediately transported to the laboratory. Then, adipose tissue was minced with scissors and digested by liberase (final concentration 15 µg/ml). Isolated adipocytes were obtained within 3h from the start of surgery. After filtration and washing as previously described [1], fat cell suspensions were diluted in the same medium as for digestion, but without liberase, i.e. Krebs – Ringer containing 15 mM sodium bicarbonate, 10 mM 4-(2-hydroxyethyl)-1-piperazineethanesulfonic acid and 3.5 g/100 mL bovine serum albumin, pH was set at 7.4 after gassing with 95% CO_2/5% O_2. The cell amount per incubation vial was equivalent to 19 ± 1 mg lipid/400 µl for glucose uptake assays (without glucose and with 2 mM pyruvate), as determined by slight modifications of the method of Atgié et al. [2].

Culture of human multipotent adipose-derived stem cells

hMADS cells were cultured and maintained in proliferation medium (DMEM low glucose 1g/l, 10% FBS, 2 mM L-glutamine, 10 mM HEPES buffer, 50 units/ml of penicillin, 50 mg/ml of streptomycin, supplemented with 2.5 ng/ml of human fibroblast growth factor 2 (FGF2)) as previously described [3]. The cells were inoculated in 6-well plates at a density of 44,000 cells/ml and kept at 37°C in 5% CO_2. Six days post-seeding, FGF2 was removed from proliferation medium. On the next day (day 0), the cells were incubated in differentiation medium (DM; serum-free proliferation medium/Ham's F-12 medium containing 10 µg/ml of transferrin, 10 nM of insulin, 0.2 nM triiodothyronine, 100 µM 3-isobutyl-1-methylxanthine, 1 µM dexamethasone and 100 nM rosiglitazone). At day 3, dexamethasone and 3-isobutyl-1-methylxanthine were omitted from DM and at day 10 rosiglitazone was also omitted. Human ANP or BNP treatment (100 nM) was carried out at day 14. Human FGF2, insulin, triiodothyronine, transferrin, 3-isobutyl-1-methylxanthine, and dexamethasone were from Sigma; L-glutamine, penicillin, and streptomycin from Invitrogen; Hepes, Dulbecco's modified Eagle medium low glucose, and Ham's F-12 medium from Lonza; and rosiglitazone from Alexis Biochemicals.

Supplementary Figures Legend

Supplementary Figure 1. Human adipose *NPR1* (A) and *NPR3* (B) mRNA levels in relation to quartiles of HOMA-IR (n=33-144 per group from cohort 1).

Supplementary Figure 2. Correlations between human adipose tissue mRNA levels of *NPR1* and adipose *MLXIPL* (A-C) and *GLUT4* (B-D) gene expression in cohort 2 (n=56) (A-B) and cohort 1 (n=323) (C-D).

Supplementary Figure 3. (A) Dose-response effect of insulin on 2-deoxyglucose uptake in presence or absence of 100 nM of ANP in human isolated adipocytes (n=13). (B) Dose-response effect of insulin on 2-deoxyglucose uptake in human isolated adipocytes from lean (n=14) versus overweight/obese subjects (n=13). ** $p<0.01$ vs. lean.

Supplementary Figure 4. mRNA levels of *NPR1*, *NPR3* and *PRKG1* (cGMP-dependent protein kinase 1) during the time-course of differentiation of hMADS adipocytes from day 0 to day 13. Data are expressed as % of day 0 (n=3).

Supplementary Figure 5. Dose-response effect of ANP (A) and insulin (B) on 2-deoxyglucose uptake in differentiated hMADS adipocytes (n=8).

Supplementary Figure 6. Representative blot and quantitative bar graph of p38 MAPK Thr180 phosphorylation relative to total p38 MAPK pan (all isoforms) in response to 20 min and 60 min treatment with BNP 100 nM in absence or presence of (Rp)-8-pCPT-cGMPS 100 µM (PKG inhibitor, PKGi). * $p<0.05$, *** $p<0.001$ vs. control (n=6).

Supplementary Figure 7. (A) BNP (100 nM) mediated glucose uptake in absence or presence of Rapamycin 100 nM (mTOR inhibitor) in differentiated hMADS adipocytes (n=8). (B) Representative blot and quantitative bar graph of Akt Ser473 phosphorylation relative to total Akt in response to 20 min and 60 min treatment with BNP 100 nM in absence or presence of Rapamycin 100 nM (mTOR inhibitor). * $p<0.05$ vs. control (n=4-6).

Supplementary Figure 8. Effect of 6h treatment with ANP and BNP 100 nM on mRNA levels of *de novo* lipogenic genes such as *ACC1*, *ELOVL6*, *FASN* and *MLXIPL* (ChREBP) in differentiated hMADS adipocytes (n=6).

Supplementary References

1. Mercader J, Iffiu-Soltesz Z, Brenachot X et al (2010) SSAO substrates exhibiting insulin-like effects in adipocytes as a promising treatment option for metabolic disorders. Future Med Chem 2:1735-49

2. Atgie C, Sauvant P, Ambid L and Carpene C (2009) Possible mechanisms of weight loss of Siberian hamsters (Phodopus sungorus sungorus) exposed to short photoperiod. J Physiol Biochem 65:377-86

3. Girousse A, Tavernier G, Valle C et al (2013) Partial inhibition of adipose tissue lipolysis improves glucose metabolism and insulin sensitivity without alteration of fat mass. PLoS Biol 11:e1001485

Supp. Figure 1

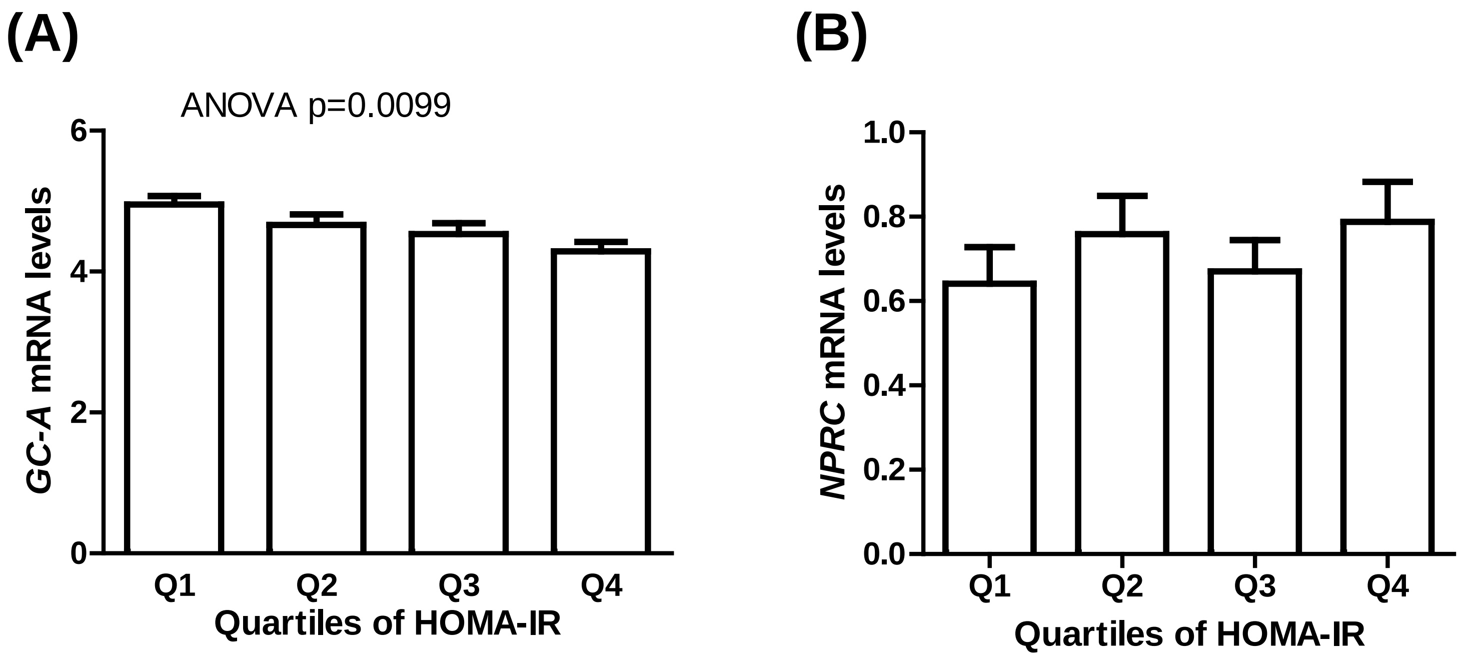

Supp. Figure 2

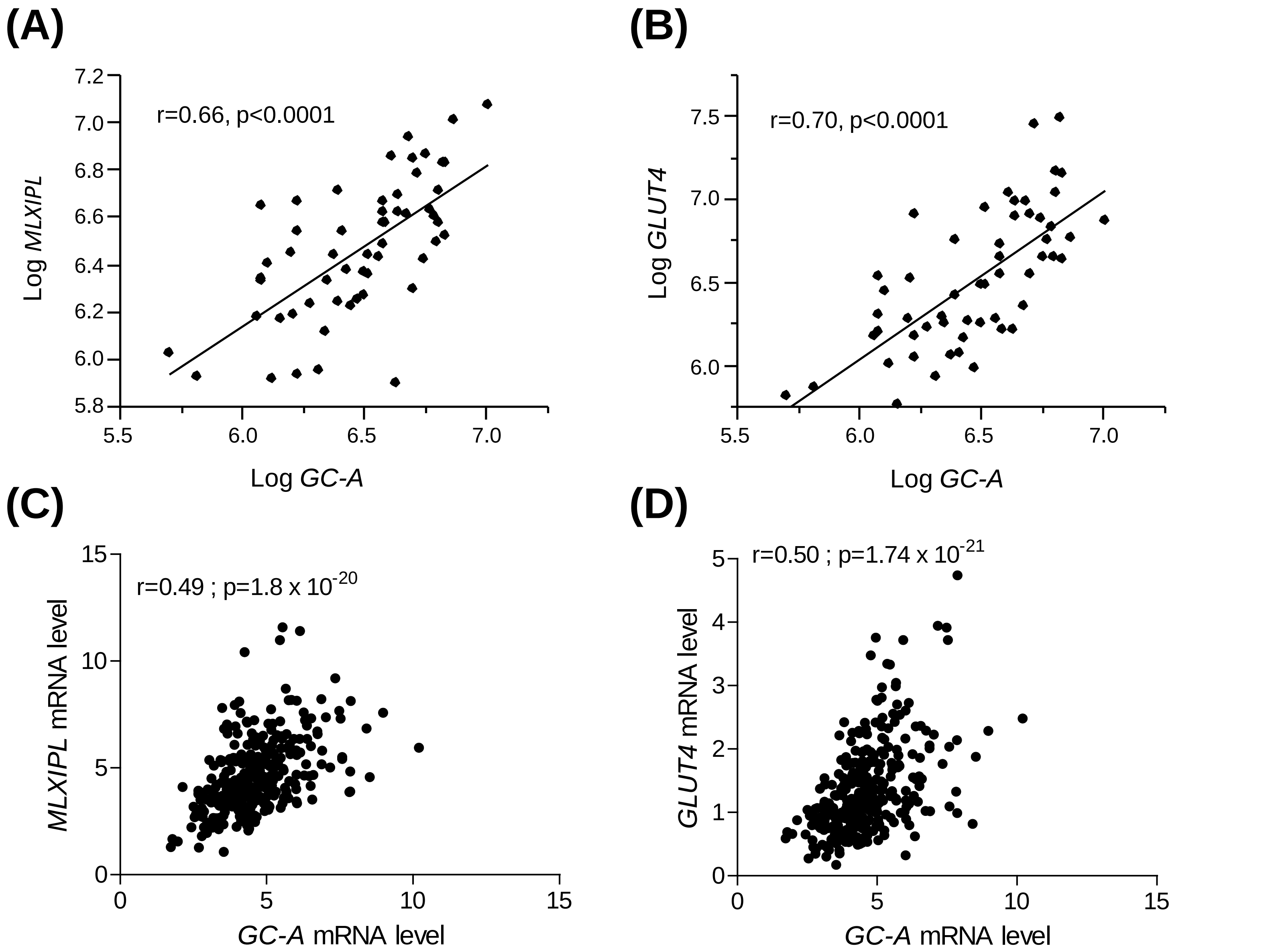

Supp. Figure 3

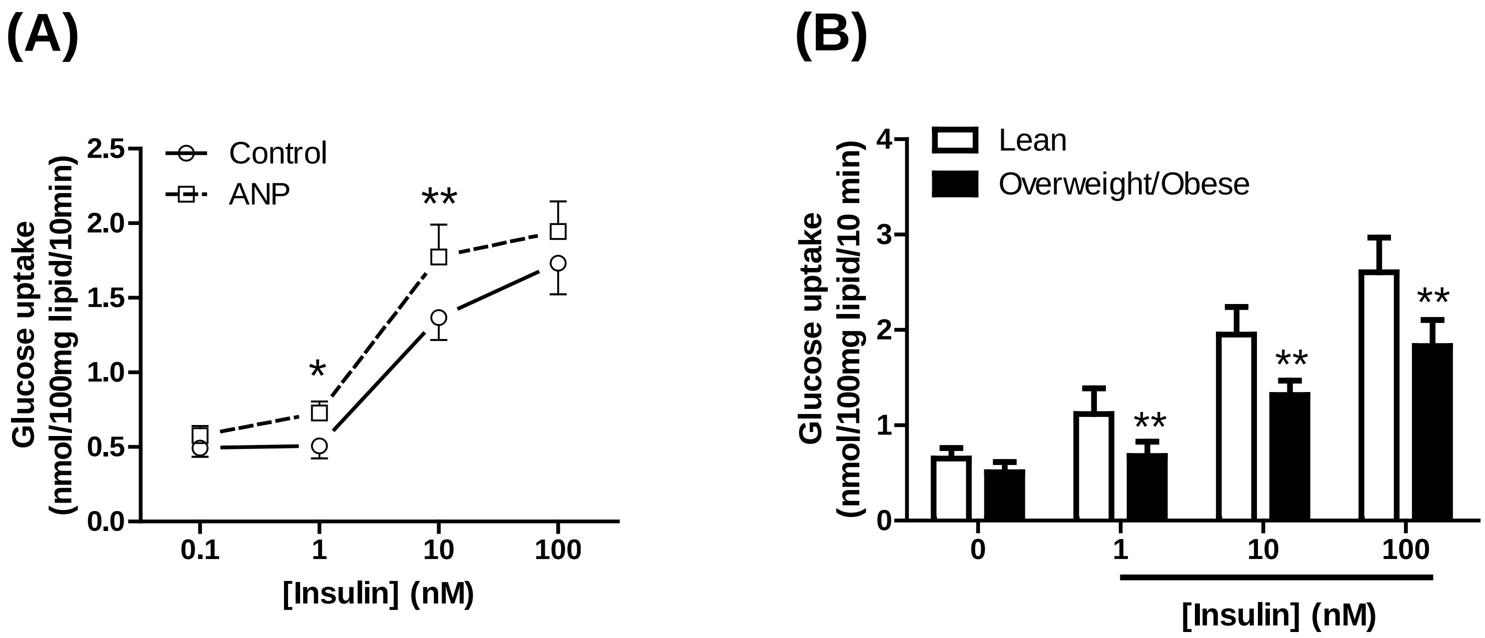

Supp. Figure 4

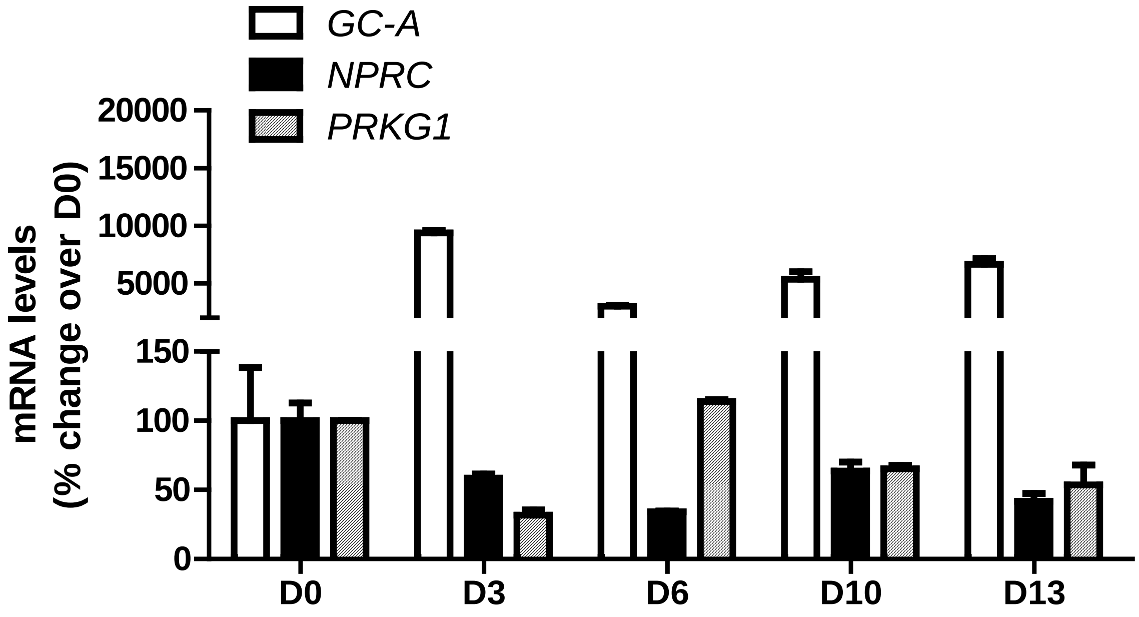

Supp. Figure 5

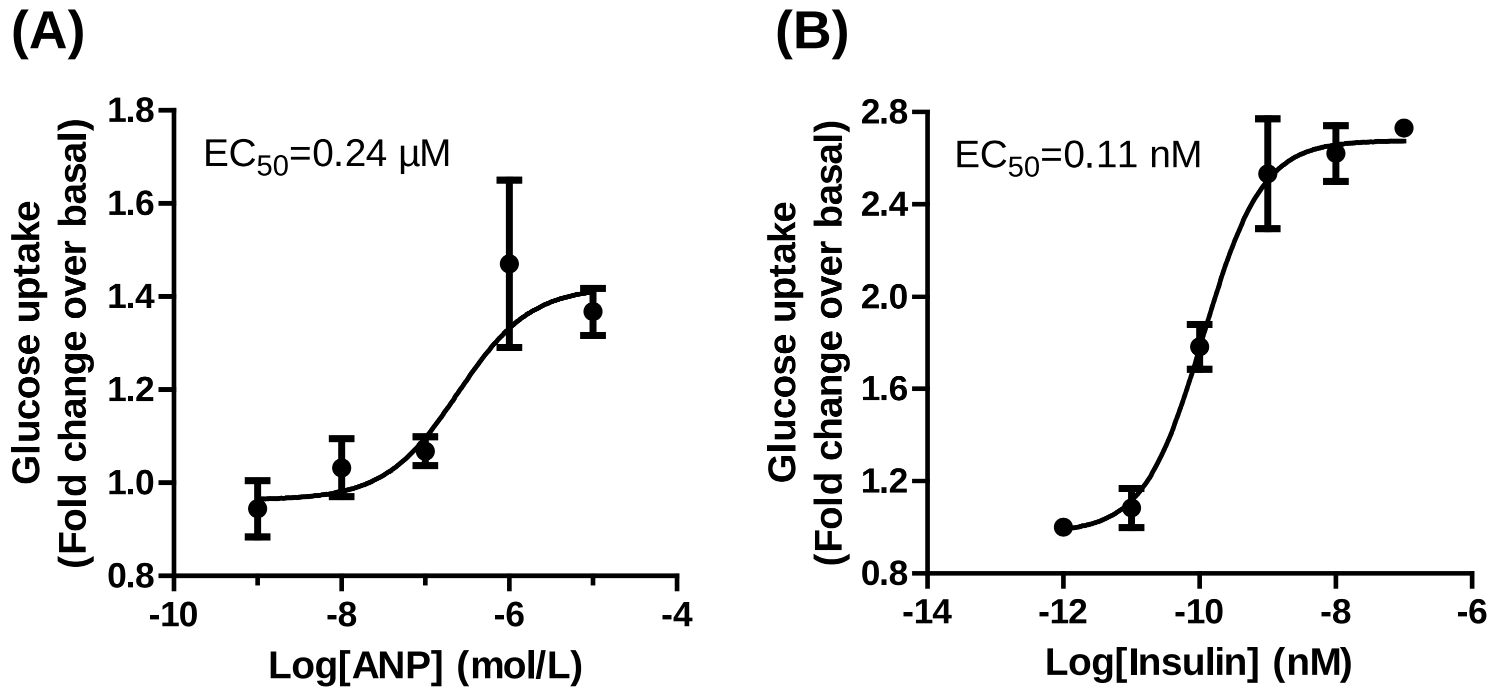

Supp. Figure 6

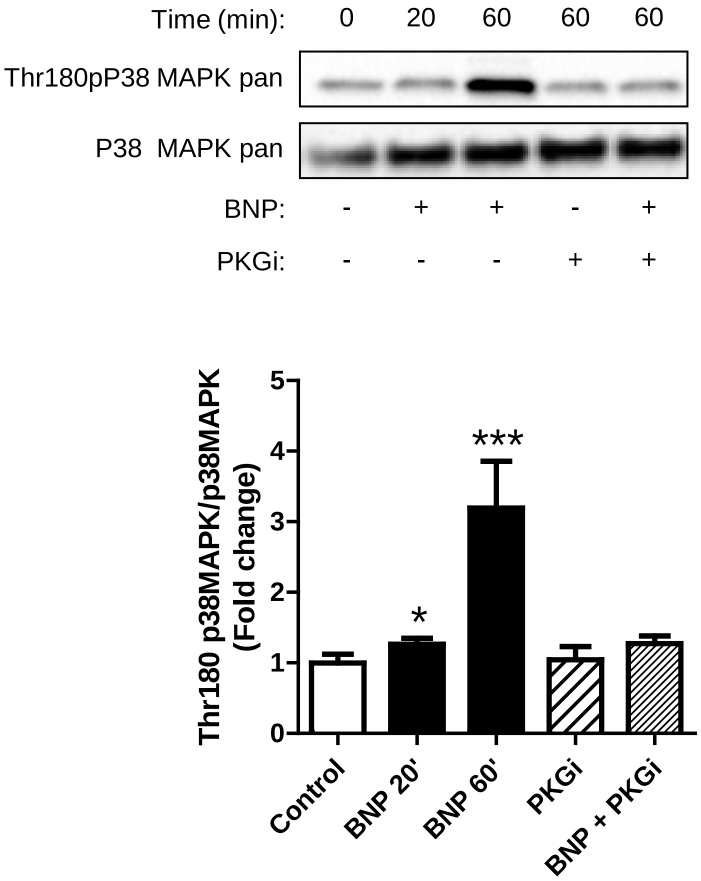

Supp. Figure 7

(A)

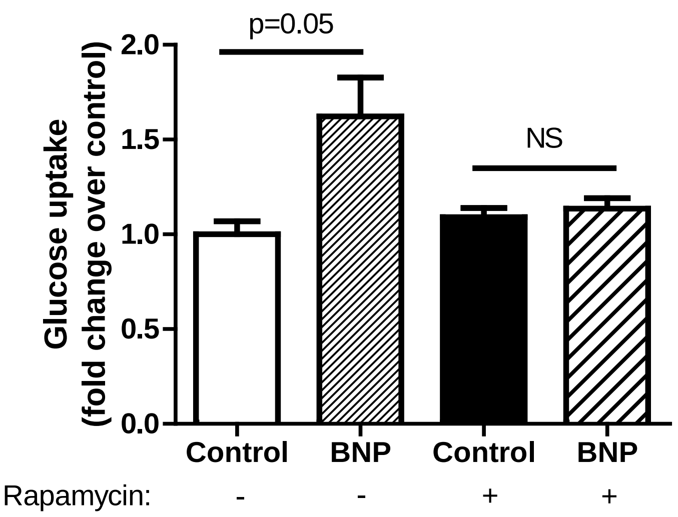

(B)

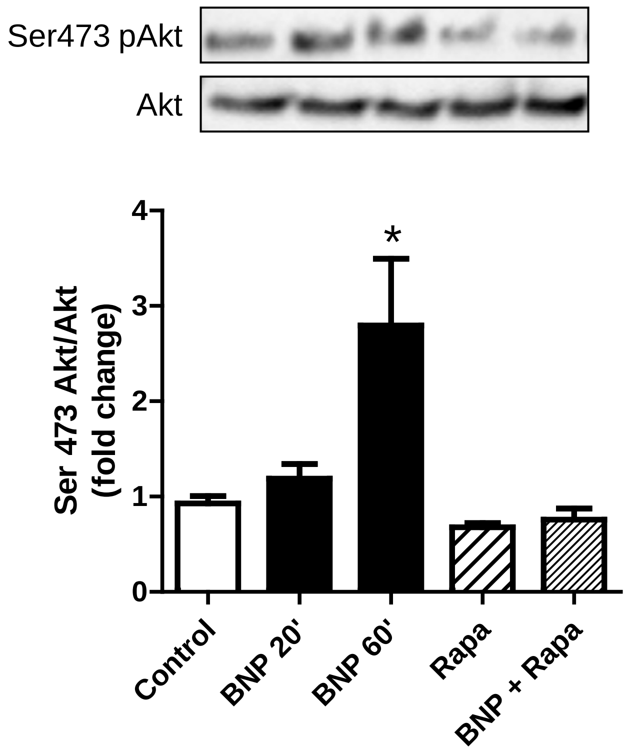

Supp. Figure 8

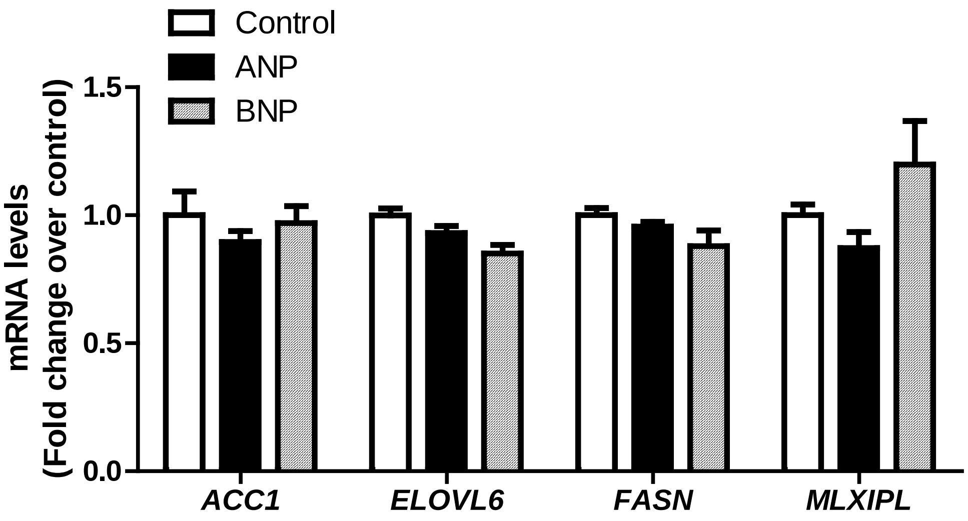

Supplement: Supplementary file 1 — Supplementary Information [file 41598_2018_19619_MOESM1_ESM.pdf]
